# The Protective Role of Mature Defense Mechanisms on Satisfaction with Life in the COVID-19 Era: A Moderated Mediation Analysis

**DOI:** 10.3390/bs12080290

**Published:** 2022-08-17

**Authors:** Alessio Gori, Eleonora Topino, Alessandro Musetti, Marco Giannini, Rosapia Lauro Grotto, Andrea Svicher, Annamaria Di Fabio

**Affiliations:** 1 Department of Health Sciences, University of Florence, Via di San Salvi 12, Pad. 26, 50135 Firenze, Italy; 2 Department of Human Sciences, LUMSA University of Rome, Via della Traspontina 21, 00193 Rome, Italy; 3 Department of Humanities, Social Sciences and Cultural Industries, University of Parma, Via M. D’Azeglio, 85, 43125 Parma, Italy; 4 Department of Education and Psychology (Psychology Section), University of Florence, Via di San Salvi 12, Complesso di San Salvi, Padiglione 26, 50135 Florence, Italy

**Keywords:** life satisfaction, anxiety, post-traumatic impairment, defence mechanism, COVID-19 pandemic, positive healthy organizations, positive strength-based preventive actions

## Abstract

The COVID-19 pandemic significantly influenced people’s lives, with non-negligible consequences for the perception of well-being. This study sought to examine the effect of anxiety, post-traumatic impairment, and mature defenses on life satisfaction during the COVID-19 pandemic. One thousand three hundred thirty-nine Italian individuals (30% male; 70% female; Mage = 34.70; SD = 11.83) completed an online survey enclosing the Satisfaction with Life Scale, State-Trait Anxiety Inventory—Form X3, Impact of event scale—revised, and the Forty Item Defense Style Questionnaire. To test the hypothesized relationship, data were analyzed by applying a moderated-mediation analysis, a regression-based approach. Results showed that the negative effect of anxiety on life satisfaction was partially mediated by post-traumatic impairment, with a significant moderation effect of mature defenses on the relationship between post-traumatic impairment and life satisfaction. Specifically, with higher levels of mature defenses, the effects of post-traumatic impairment on consequences of the COVID-19 emergency on well-being. Furthermore, the protective role of mature defenses in facing post-traumatic impairment was shown. Such data may have applicative implications in different contexts in a management perspective of the different pandemic phases, contributing to more effective positive strength-based preventive actions to also support positive healthy organizations.

## 1. Introduction

The COVID-19 pandemic is a health emergency that has rapidly involved the whole globe, leading to an escalation of interventions, preventive measures, and phases aimed at protecting the physical health of the world population [1]. This resulted in substantial changes in the community and individuals’ lives, which generated high degrees of job insecurity and significant levels of distress, thus making the pandemic not only a medical problem but also an economic, social crisis and, above all, a risk for well-being of people [2]. Indeed, the spread of COVID-19 and related events caused a variety of psychological impairments [3] and had a negative effect on perceptions concerning quality and satisfaction of life [4,5], including in work environments [6,7]. Given this framework, the present study aimed to examine the factors that may influence life satisfaction during the COVID-19 pandemic, specifically exploring the roles of anxiety, post-traumatic impairment, and mature defenses in this relationship.

### 1.1. The Association between Anxiety and Satisfaction with Life

Satisfaction with life is a key component of subjective well-being and could be defined as a general assessment of the individual regarding his own life, linked to the extent to which his/her needs and desires are satisfied, and his/her goals achieved [8]. It is associated with resilience [9], self-esteem [10], and positive affects [11]. Furthermore, previous studies showed its protective role against perceived stress associated with the situation of the present pandemic by favoring the use of functional strategies for better adaptation [12]. Differently, lower levels of satisfaction with life have been related to depression, suicide attempts, addictions, or anxiety [13]. Indeed, among the dimensions influencing life satisfaction, anxiety is one of the most reported during the pandemic: Wang and colleagues [14] showed that about one-third of the participants in the general Chinese population declared moderate to severe anxiety without significant longitudinal changes in those levels. This, in turn, may lead to a series of cascade reactions that could negatively affect life satisfaction [4,14], as demonstrated by previous research indicating the association between anxiety and functional impairment, unhealthy coping strategies, hopelessness [15,16], and lower quality of life [17]. Therefore, based on the above empirical evidence, hypothesis 1 is developed as follows:

**H1.**  *Anxiety will be negatively associated with satisfaction with life.*

### 1.2. The Mediating Role of Post-Traumatic Impairment

Post-traumatic stress disorder (PTSD) encloses a problematic impairment that may be generated by terrifying events perceived as outside normal human experiences [18]. In this regard, the COVID-19 pandemic was an unexpected and frightening event [19] that generated high levels of instability and uncertainty [20], stress [21], and general psychopathological symptomatology [22]. Indeed, anxiety due to the perceived threat of COVID-19 can be a relevant predictor of it [23], and several studies highlighted a tendency to report post-traumatic symptoms impairments linked to the pandemic, which could persist even when the emergency is over [4,14]. Furthermore, PTSD may be associated with worsening physical health functioning, psychosocial impairments, and lower subjective quality of life (see Holowka and Marx, [24] for a review), as well as the lower perception of life satisfaction [25]. Therefore, based on the above empirical evidence, hypothesis 2 is developed as follows:

**H2.**  *Post-traumatic impairment will mediate the relationship between anxiety and satisfaction with life*.

### 1.3. The Moderating Effect of Mature Defences

Post-traumatic impairment due to potentially traumatizing events does not certainly lead to long-lasting negative impacts on the life of individuals, but on the contrary, they may be transformed into a new form of adaptation [26]. The psychological responses to stressful events may be influenced by the ability to appropriately use defense mechanisms [27], defined as processes that can influence individual reactions to internal or external stressors [28]. In this regard, while maladaptive defensive functioning could be linked to a large variety of psychological impairments, unhealthy behaviors and a wide array of disorders [29], a mature defense style was found to be a protective factor against psychological symptoms [30]. Indeed, mature defences can be defined as mechanisms that correctly “*integrate reality, interpersonal relationships, and private feelings*” [31] (p. 247) and are significantly and positively associated with subjective well-being and satisfaction with life [32]. Therefore, based on the above empirical evidence, hypothesis 3 is developed as follows:

**H3.**  *Mature defences will moderate the association between post-traumatic impairment and* *satisfaction with life.*

## 2. Materials and Methods

### 2.1. Participants and Procedures

The current research was run on 1339 Italian individuals, of which 937 were women and 402 were men, aged between 18 and 88 years (*Mage* = 34.70; *SD* = 11.83). All participants were recruited with a snowball procedure on the Internet and filled out an anonymous web survey through the Google Forms platform. They were briefed on the overall purposes of the study and provided electronically informed consent before starting. The inclusion criteria were a minimum age of 18 years and to declare of being Italians living in Italy at the time of administration. Each respondent voluntarily participated in the study without receiving compensation for his involvement and was free to leave the research at any moment. The survey was launched on 20 March 2020 and remained open until 29 March 2020 (a period corresponding to 10 days during the Italian National Lockdown). The administrations were carried out according to the Italian privacy laws (Law Decree DL-196/2003) and European Union General Data Protection Regulation (EU 2016/679). The study was approved by the Ethical Committee of the Integrated Psychodynamic Psychotherapy Institute (IPPI; ethical approval number 004/2020).

### 2.2. Measures

#### 2.2.1. Satisfaction with Life Scale (SWLS)

The Satisfaction with Life Scale (SWLS) [33]—Italian version [34,35] was used to assess the overall level of satisfaction with life in respondents. The SWLS is a 5-item self-administered questionnaire assessed through a seven-point Likert scale, from 1 (=“*Strongly disagree*”) to 7 (=“*Strongly agree*”). The scale has reported psychometrically sound properties in previous research among Italian workers [35], showing a Cronbach alpha (α) of 0.85.

#### 2.2.2. State-Trait Anxiety Inventory—Form X3 (STAI-X3)

The State-Trait Anxiety Inventory—Form X (STAI-X) [36] was used in its short Italian Version [37,38]. This brief version is focused on the general assessment of the levels of state anxiety only [37,38]. It has 10 items ranked on a 4-point Likert scale from 1 (=“*Not at all*”) to 4 (=“*Very much so*”). The short Italian version showed good psychometric properties in a past study run on healthy subjects (α = 0.90). [38].

#### 2.2.3. Impact of Event Scale—Revised (IES-R)

The Impact of event scale—revised (IES-R) [39] was administered in its Italian version [40] to assess the respondent’s post-traumatic impairment. The IES-R is a 22-item self-report questionnaire composed of three dimensions with eight items each, namely Intrusion, Avoidance, and Hyperarousal. The Italian scale showed satisfactory psychometric properties in its validation study, with good internal consistency in all the subscales (intrusion, α = 0.78; avoidance, α = 0.72; hyperarousal, α = 0.83) [40].

#### 2.2.4. Forty Item Defense Style Questionnaire (DSQ-40)

The Forty Item Defense Style Questionnaire (DSQ-40) [41] was used in its Italian version [42] to assess the respondent’s defense mechanisms. The DSQ-40 is a 40-item self-report tool with each item ranked on a nine-point Likert scale, from 1 (=“*Strongly disagree*”) to 9 (=“*Strongly agree*”). The authors of the Italian versions confirmed the acceptable psychometric properties of the measure [42], which assesses three main styles: (1) The mature defense style (sublimation, humor, anticipation, and suppression; α = 0.61), (2) the neurotic defense style (pseudo-altruism, idealization, and reaction formation; α = 0.59), (3) the immature defense style (projection, acting out, isolation, devaluation, autistic fantasy, denial, passive aggressiveness, displacement, disassociation, splitting, rationalization, and somatization, α = 0.80) [42]. In the current study, the mature defense style score was used.

### 2.3. Analytic Plan

Data analysis was run by implementing the SPSS statistical software (v. 25.0, IBM, Armonk, NY, USA). First, the inspection of Person’s correlation was run to examine the association between the study variables. To investigate the relationship between anxiety and satisfaction with life, and also explore the role of post-traumatic impairment and the influence of mature defense mechanisms, a moderated mediation model (Model 14) was implemented by using the macro-program PROCESS 3.4 [43]. For each regression coefficient calculated in the model, the 95% confidence interval (CI) was provided. The conditional indirect effect was further assessed through the Johnson–Neyman technique [44], showing how the effect of a predictor on an outcome varies from being significant or not based on the value of the moderator. Therefore, following the Wayne et al. [45] procedure, the magnitude of the interaction was tested by exploring the conditional effects of post-traumatic impairment symptoms at three levels of mature defense style, i.e., −1 SD Mean, +1 SD. Furthermore, the statistical significance was of the moderated mediation model was further explored by using the Bootstrap technique (5000 bootstrapped samples with 95% CI), which indicates its significance when the CI (from Lower Level of Confidence Interval [LLCI] to Upper Level of Confidence Interval [ULCI]) does not contain zero. Finally, the moderated mediation analysis was also replicated by controlling confounders (i.e., age, gender) in the models.

## 3. Results

As shown in Table 1, Pearson’s correlation coefficients highlighted significant and negative associations between anxiety and satisfaction with life (*r* = −0.314, *p* < 0.01). Furthermore, anxiety was also positively and significantly associated with post-traumatic impairment (*r* = −0.601, *p* < 0.01), which, in turn, was negatively and significantly related with satisfaction with life (*r* = −0.212, *p* < 0.01). Finally, mature defenses showed significant correlations with Satisfaction with life (*r* = 0.202, *p* < 0.01) and Anxiety (*r* = −0.161, *p* < 0.01).

**Figure 1:**
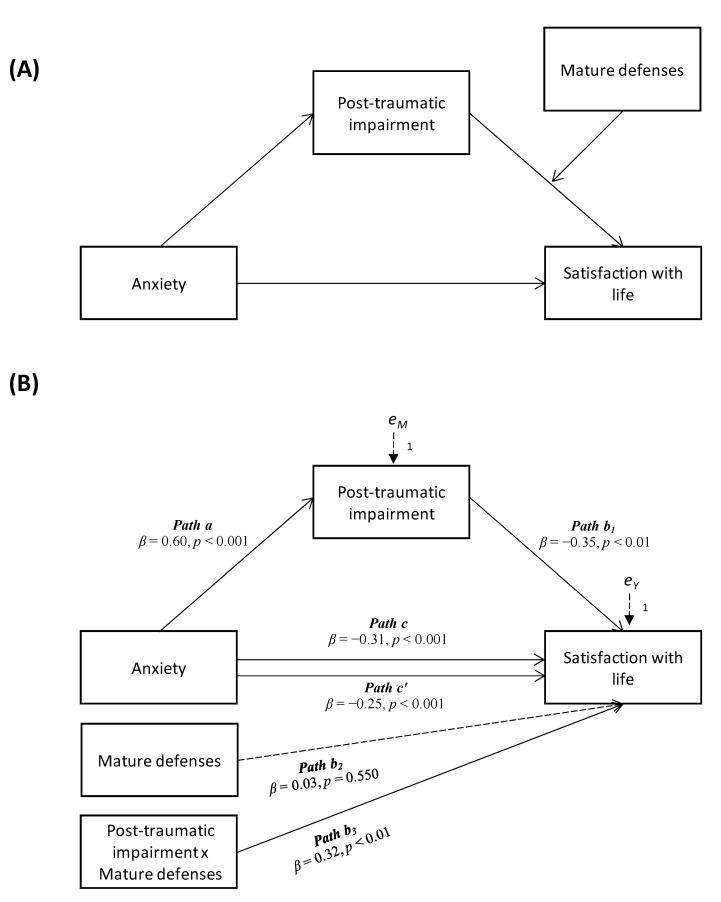
Conceptual (**A**) and statistical (**B**) form of a moderated mediation model enclosing anxiety, post-traumatic impairment, mature defenses and satisfaction with life.

The results of the moderated mediation analysis displayed a statistically significant negative effect of anxiety on satisfaction with life, with the mediation of post-traumatic symptoms impairment, the effect of which is, therefore, moderated by the use of mature defenses (see Figure 1).

Specially, anxiety was negatively and significantly associated to satisfaction with life (Path *c* in Figure 1B; *β* = −0.31, *p* < 0.001, LLCI = −0.3236–ULCI = −0.2334), and significantly and positively related to post−traumatic symptoms impairment (Path *a* in Figure 1B; *β* = 0.60, *p* < 0.001, LLCI = 1.1947–ULCI = 1.3781), which partially mediated and reduced the effect of anxiety on satisfaction with life when included in the model (Path *c’* in Figure 1B; *β* = −0.25, *p* < 0.001, LLCI = −0.2799 –ULCI = −0.1666): *R*^2^ = 0.129, *F*(4, 1334) = 49.571, *p <* 0.001. Indeed, as showed in Table 2 (Model 1), post-traumatic symptoms impairment had a statistically significant and positive effect on satisfaction with life (Path *b*_1_ in Figure 1B; *β* = −0.35, *p* < 0.01, LLCI = −0.2330–ULCI = −0.0547), although with the moderation of mature defense mechanisms (Path *b*_3_ in Figure 1B; *β* = 0.32, *p* < 0.01, LLCI = 0.0008–ULCI = 0.0047): Index = 0.0036, Boot LLCI = 0.0007–Boot ULCI = 0.0060.

**Figure 2:**
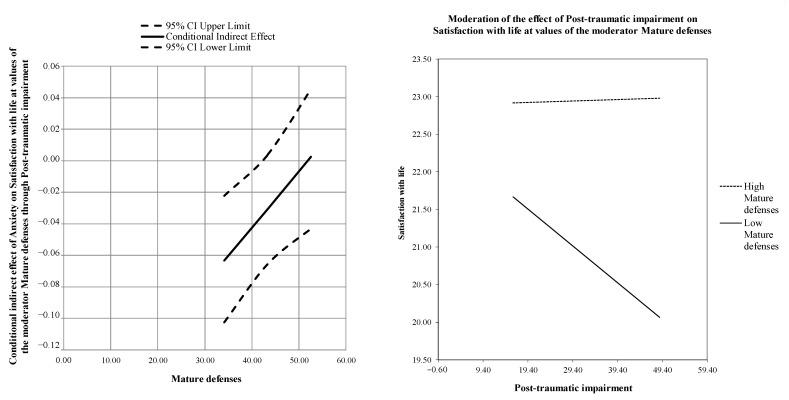
Johnson–Neyman plot and graphic representation of the moderated effect.

The conditional indirect effect was further assessed through the Johnson–Neyman technique [44] and the Wayne et al. [45] procedure. The negative effect of post-traumatic impairment symptoms on satisfaction with life was significant at low levels of mature defences (estimate = −0.05(0.02), *p* < 0.01; LLCI = −0.0808; ULCI = −0.0174), but not at average (estimate = −0.02(0.01), *p* = 0.077; LLCI = −0.0497; ULCI = 0.0025) and high levels (estimate = −0.00(0.02), *p* = 0.908; LLCI = −0.0300; ULCI = 0.0338). Thus, when participants showed higher and average levels of mature defenses, the negative indirect effect of anxiety on satisfaction with life through post-traumatic symptoms impairment became insignificant (see Figure 2). Finally, the Bootstrap analysis confirmed that the moderation effect was relevant and robust: Boot LLCI = 0.001–Boot ULCI = 0.005.

The effect of potential confounders (i.e., age and gender) was also examined, and the relationships highlighted in the model maintained their significance, further identifying their statistical solidity (see Model 2 in Table 2).

## 4. Discussion

The COVID-19 pandemic profoundly altered the everyday life of people all around the globe, with significant effects not only on individuals’ well-being [46,47] but also on the well-being of workers and organizations [25,48]. Furthermore, the current pandemic has negatively affected subjective life satisfaction, which, for its part, proved to be an important protective factor for individual well-being during this health emergency [12], particularly challenging also in the framework of positive healthy organizations [6,49].

Starting from these premises, the current research aimed to better understand the factors that may influence this important dimension and aimed therefore to deepen the factors that may influence satisfaction with life during the pandemic, specifically considering the effects of anxiety, post-traumatic impairment, and mature defenses.

### 4.1. The Association between Anxiety and Satisfaction with Life

Results showed a significant and negative link between anxiety and life satisfaction, supporting the first hypothesis (H1). This is in accordance with past research highlighting that poorer mental health and, more specifically, higher anxiety levels were related to lower perceptions of life satisfaction [13]. This information could be read considering that a worsening in quality of life may be linked to higher anxiety levels resulting from the pandemic [50] which, in turn, could be associated with a decline in perceived well-being and, more specifically, in the sense of satisfaction with life [51].

### 4.2. The Mediating Role of Post-Traumatic Impairment

Results also highlighted an indirect path in the relationship between anxiety and satisfaction with life, suggesting the mediating effect of post-traumatic symptoms impairment in this association and confirming the second hypothesis (H2). These results are consistent with previous findings, suggesting that the current pandemic has determined high levels of anxiety, which may promote the development of post-traumatic impairment [52,53]. This, in turn, was related to worse physical health conditions and lower quality of life [54]. In the presence of such a disturbing event as a global health crisis affecting every area of life, indeed, manifestations of distress may be common [55].

### 4.3. The Moderating Effect of Mature Defences

The psychological consequences of the event may be influenced by the personal use of defense mechanisms [56]. This was supported by the findings of current research, which confirmed the third hypothesis (H3) by showing moderation in the indirect relationship between anxiety and life satisfaction, specifically in the effect that post-traumatic impairment had on the latter. In the indirect path, for individuals who use higher levels of mature defenses, the effects of post-traumatic symptoms impairment on life satisfaction lose their strength and significance. This was supported by previous evidence showing that not all people exposed to traumatic events report persistent psychological impairment [57,58]: Some may experience deterioration in functioning, gradually returning to a state of satisfactory adaptation, while others also maintain significant psychological comorbidities (e.g., [26,59]). Indeed, mature defense mechanisms may operate as processes that maximize gratification and awareness of the subject’s real feelings [55], favoring better psychophysical health [56,60] and subjective well-being [32], consistently with the data of this study, where the negative effect of the post-traumatic impairment on satisfaction with life was significant only for average or low levels of mature defenses.

### 4.4. Limitations and Suggestions for Future Research

These findings should be cautiously interpreted due to some limits that should be addressed. First, data were collected online by spreading an anonymous link through a snowball procedure: This may have excluded a portion of the population (e.g., those who did not have internet access) and, therefore, the participants recruited in this study may not be representative of the general population. Furthermore, the low Cronbach’s alpha of the mature defenses scale should be considered in interpreting these results. Indeed, although values of 0.60 may be considered acceptable [61], such findings should be replicated in future research by using measures with higher internal consistency. Moreover, only self-report measures were used: This exposes the risk of several biases (e.g., that of social desirability), which could limit the veracity of the information obtained. Future research could overcome these limitations by integrating different methods of recruiting and data collection, e.g., also using structured interviews in a one-to-one setting. Then, this research implemented a cross-sectional design that impedes definitively establishing causal inferences in the relationship between the variables involved in the applied model. In future research, a longitudinal approach could help to give further evidence, deepening and extending these results. Finally, this study did not explore the participants’ socioeconomic status (SES) or education levels, which could impact satisfaction with life during the pandemic [62]. Future research could integrate and expand the given findings by exploring these aspects as well to give a more accurate framework of the psychological consequences of the COVID-19 emergency.

### 4.5. Practical Implications

Despite the limitations, this study also proposes novel aspects with valid practical implications. The exploration of the role of anxiety, post-traumatic impairment, mature defences, and their mutual interactions in influencing satisfaction with life offers important information that contributes to enriching the existing literature in this field [3,12,15,27,46]. Although the data were collected during the COVID-19 pandemic, they offer important application insights that can be useful both for emergency management and outside of it. The analysis of the relationships between anxiety, post-traumatic impairment, and satisfaction with life also highlights the importance of developing adequate interventions to manage distress in various life situations and contexts. In this regard, these results suggest that implementing interventions aimed at the correct management of anxiety [63] and post-traumatic impairment [64] can limit the negative effect of these variables on life satisfaction. In the same line, the importance of acquiring mature and adequate defensive mechanisms was also highlighted. These results could also give some insights into other contexts, opening promising lines of research, also in the field of positive healthy organization [6] in which mature defense mechanisms could be part of positive psychological resources in need to be promoted for healthy workers and healthy businesses. Furthermore, since mature defense mechanisms seem to be related to a large array of positive outcomes and were found able to ameliorate the relationship between post-traumatic impairment and anxiety, they could be promising for the perspective of positive preventive strength-based actions [65,66,67] in the everyday life and the work contexts.

## 5. Conclusions

The findings of this study highlighted the relationship between anxiety, post-traumatic impairment, mature defenses, and life satisfaction at the time of the COVID-19 pandemic. The deepening of the responses to this emergency is an important topic, given the effect that these traumatic events may have on psychological well-being and in producing interference with people’s life. Indeed, the COVID-19 crisis is not only a medical and economic problem, but several researchers also highlighted a significant and negative influence on the perception of life satisfaction [4,5] on well-being [47] and organizational well-being and functioning [68].

More specifically, the results of this research highlighted that the levels of anxiety during the pandemic were negatively associated with life satisfaction, both with a direct and indirect path, involving in the latter the effect of the post-traumatic impairment. However, this indirect path appeared to be characteristic of those subjects with less adaptive defensive functioning. Indeed, defense mechanisms could have an important role in facing traumatic events and may have a key role in stress adaptation or distress development [69]. In other words, the findings support the use of mature defenses as a protective factor for life satisfaction during the COVID-19 pandemic, at least partially, by influencing the significance of the effects in the indirect path. Therefore, this study contributed to a better understanding of the variables related to psychological impairment and their relationships during the pandemic. This may have interesting practical implications in enriching interventions to favor the well-being of the population, as well as of the workers in the organizations, suggesting the relevance of increasing and supporting the use of mature defenses to face stressful events, such as the progression of the COVID-19 emergency. Furthermore, the assessment of mature defenses as a protective factor against psychological and post-traumatic impairment could be promising also in the framework of positive healthy organizations [6] and positive strength-based preventive actions [65,66,67,70,71]. Therefore, these data may have value in a management perspective of the different pandemic phases and associated consequences in several contexts, favoring the integration of the existing literature and contributing to building more effective preventive practices at an individual and organizational level.

## Figures and Tables

**Table 1:** Zero-order Pearson’s correlation matrix.

	Satisfaction with Life	Anxiety	Mature Defenses	Post-Traumatic Impairment
Satisfaction with life	1			
Anxiety	−0.314 **	**1**		
Mature defenses	0.202 **	−0.161 **	1	
Post-traumatic impairment	−0.212 **	0.601 **	−0.008	1

Note: Bold values indicate significant *p*-values. ** Correlation is significant at the 0.01 level (2-tailed).

**Table 2:** Coefficients of the moderated mediation model.

Model 1											
Antecedent	Consequent										
	M						Y				
		*B*	SE	*P*	*95% CI*		*B*	SE	*P*	*95% CI*	Test(s) of Highest Order Unconditional Interaction(s):
X	*a*	1.286	0.047	<0.001	[1.195; 1.378]	*c’*	−0.223	0.029	<0.001	[−0.280; −0.167]	
M		-	-	-	-	*b*_1_	−0.144	0.045	<0.01	[−0.233; −0.055]	
W		-	-	-	-	*b*_2_	0.024	0.039	0.550	[−0.054; 0.101]	
M × W		-	-	-	-	*b*_3_	0.003	0.001	<0.01	[0.001; 0.005]	Δ*R*^2^ = 0.005 *F*(1, 1334) = 7.621, *p* < 0.01
Constant	*i_M_*	5.287	1.048	<0.001	[3.231; 7.344]	*i_Y_*	26.354	1.804	<0.001	[22.815; 29.893]	
	*R*^2^ = 0.362 *F*(1, 1337) = 756.935, *p* < 0.001					*R*^2^ = 0.129 *F*(4, 1334) = 49.571, *p* < 0.001					
**Model 2**											
**Antecedent**	**Consequent**										
	**M**						**Y**				
		***B***	**SE**	***P***	***95% CI***		***B***	**SE**	***P***	***95% CI***	**Test(s) of Highest Order Unconditional Interaction(s):**
X	*a*_1_	1.253	0.048	<0.001	[1.159; 1.347]	*c’*	−0.220	0.029	<0.001	[−0.277; −0.163]	
M		-	-	-	-	*b*_1_	−0.148	0.045	<0.01	[−0.237; −0.059]	
W		-	-	-	-	*b*_2_	0.026	0.039	0.508	[−0.051; 0.103]	
M × W		-	-	-	-	*b*_3_	0.003	0.001	<0.01	[0.001; 0.005]	Δ*R*^2^ = 0.005 *F*(1, 1332) = 7.613, *p* < 0.01
C1	*a*_2_	0.023	0.31	0.457	[−0.037; 0.082]	*b*_4_	0.027	0.015	0.068	[−0.002; 0.056]	
C2	*a*_3_	3.560	0.790	<0.001	[2.010; 5.111]	*b*_5_	0.900	0.388	<0.05	[0.140; 1.660]	
Constant	*i_M_*	−0.838	1.923	0.663	[−4.611; 2.934]	*i_Y_*	23.865	1.975	<0.001	[19.991; 27.739]	
	*R*^2^ = 0.372 *F*(3, 1335) = 263.123, *p* < 0.001					*R*^2^ = 0.136 *F*(4, 1332) = 34.788, *p* < 0.001					

Note: Model 1: the mediation of post-traumatic impairment in the relationship between anxiety and satisfaction with life, moderated by mature defenses; Model 2 = the mediation of post-traumatic impairment in the relationship between anxiety and satisfaction with life, moderated by mature defenses, and controlling for age and gender; X = Anxiety; M = Post-traumatic impairment; W = Mature defenses; Y = Satisfaction with life. C1 = Age; C2 = Gender (coded as 1 = Men; 2 = Women).

## Data Availability

The data presented in this study are available on request from the corresponding author.
